# Supplementary material for: Incorporating simulated spatial context information improves the effectiveness of contrastive learning models
Source: Patterns (N Y). 2024 Mar 26;5(5):100964. doi: 10.1016/j.patter.2024.100964 (PMC11117056; doi:10.1016/j.patter.2024.100964)
Supplement: Document S1. Supplemental experimental procedures, Figures S1–S4, and Tables S1–S4 [file mmc1.pdf]

Patterns, Volume *5*

Supplemental information

Incorporating simulated spatial context information improves the effectiveness of contrastive learning models

Lizhen Zhu, James Z. Wang, Wonseuk Lee, and Brad Wyble

Supplemental Experimental Procedures

Room boundaries in two environments

The Archviz House ('House') consists of eight distinct rooms, with example images depicted in Figure S1. These rooms are the kitchen, lower hall, lower bedroom, outer deck, upstairs piano room, bathroom, and upstairs bedroom. Images are classified based on their position inside the defined boundaries of each room. Certain images, such as those captured on the stairs, do not fall within the boundaries of any room and are therefore excluded from the evaluation. In House14K, 12,127 of 14,766 images are labeled. In House100K, 83,300 of 102,197 images are labeled. The boundaries and the number of samples for each room are listed in Table S1.

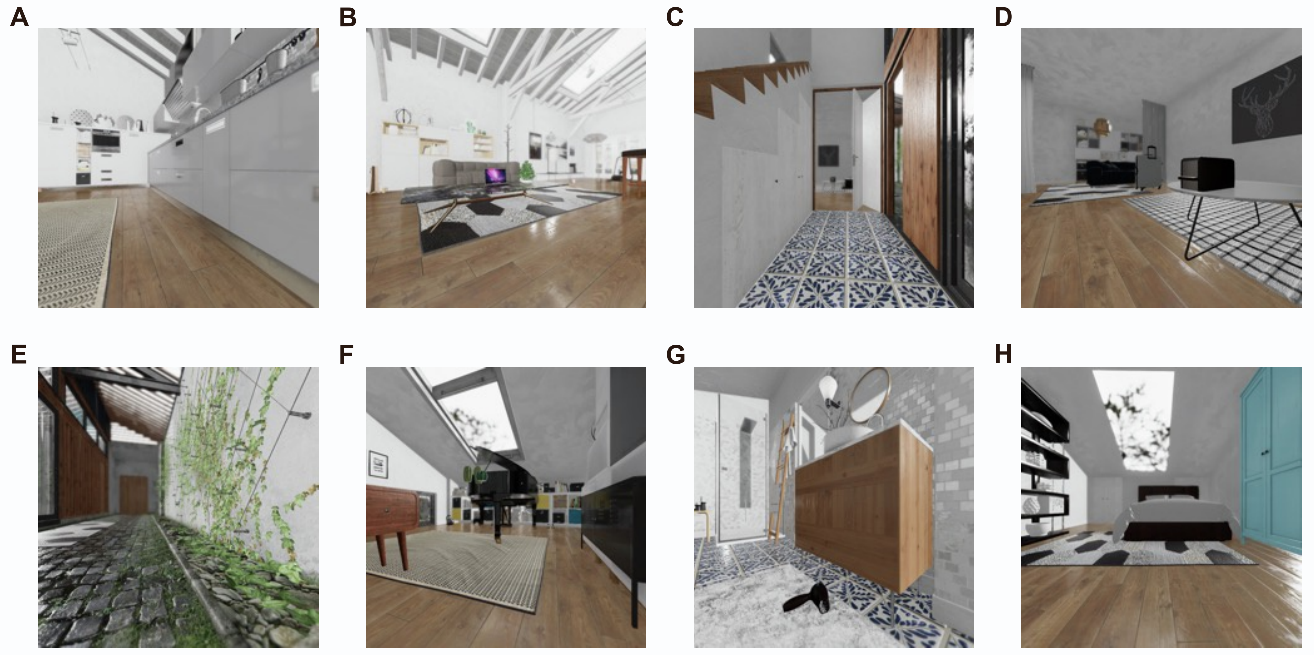

Figure S1: Eight rooms in the House environment
(A) Kitchen. (B) Living room. (C) Lower hall. (D) Lower bedroom. (E) Outer deck. (F) Upstairs piano room. (G) Bathroom. (H) Upstairs bedroom.

Room name	x-axis boundary	y-axis boundary	Height boundary	House14K images	House100K images
kitchen	(-17.00, -11.83)	(-1.48, 1.80)	(1.4, 3.5)	2079	7535
living room	(-17.00, -7.60)	(-7.00, -1.48)	(1.4, 5.2)	3225	32412
lower hall	(-6.30, -4.10)	(-4.30, -3.10)	(0.6, 3.5)	461	3490
lower bedroom	(-3.43, 0.05)	(-4.30, 1.60)	(0.6, 3.5)	1506	10155
outer deck	(-7.10, 6.00)	(-7.40, -4.75)	(0.4, 5.2)	2491	6721
upstairs piano room	(-3.20, -0.25)	(-4.30, -3.00)	(3.5, 5.2)	925	9882
bathroom	(-3.20, -0.25)	(-4.30, -3.00)	(3.5, 5.2)	419	2853
upstairs bedroom	(0.00, 4.10)	(-4.30, 1.37)	(3.5, 5.2)	1021	10252

Table S1: **The boundaries of the eight rooms in the House environment and the number of samples for each room**
The coordinate ranges are measured in the ThreeDWorld virtual environment. "House14K images" and "House100K images" means the number of images in each category for House14K and House100K respectively.

The Apartment ('Apt') layout consists of nine rooms, arranged in two rows. Rooms in the upper row in the floor plan are marked as rooms 0 to 4, from left to right. Rooms 5 to 8 are in the lower row in the same left-to-right sequence. The items placed in each room are carefully designed. For instance, entertainment facilities are filled in room 0. Rooms 3, 4, 6, and 7 serve as living rooms, each having a distinct style. Room 8 is a kitchen. In

Apt14K, 9,855 of 14,487 images are labeled. Sample images and the specified boundaries for each room are shown in Figure S2 and Table S2, respectively.

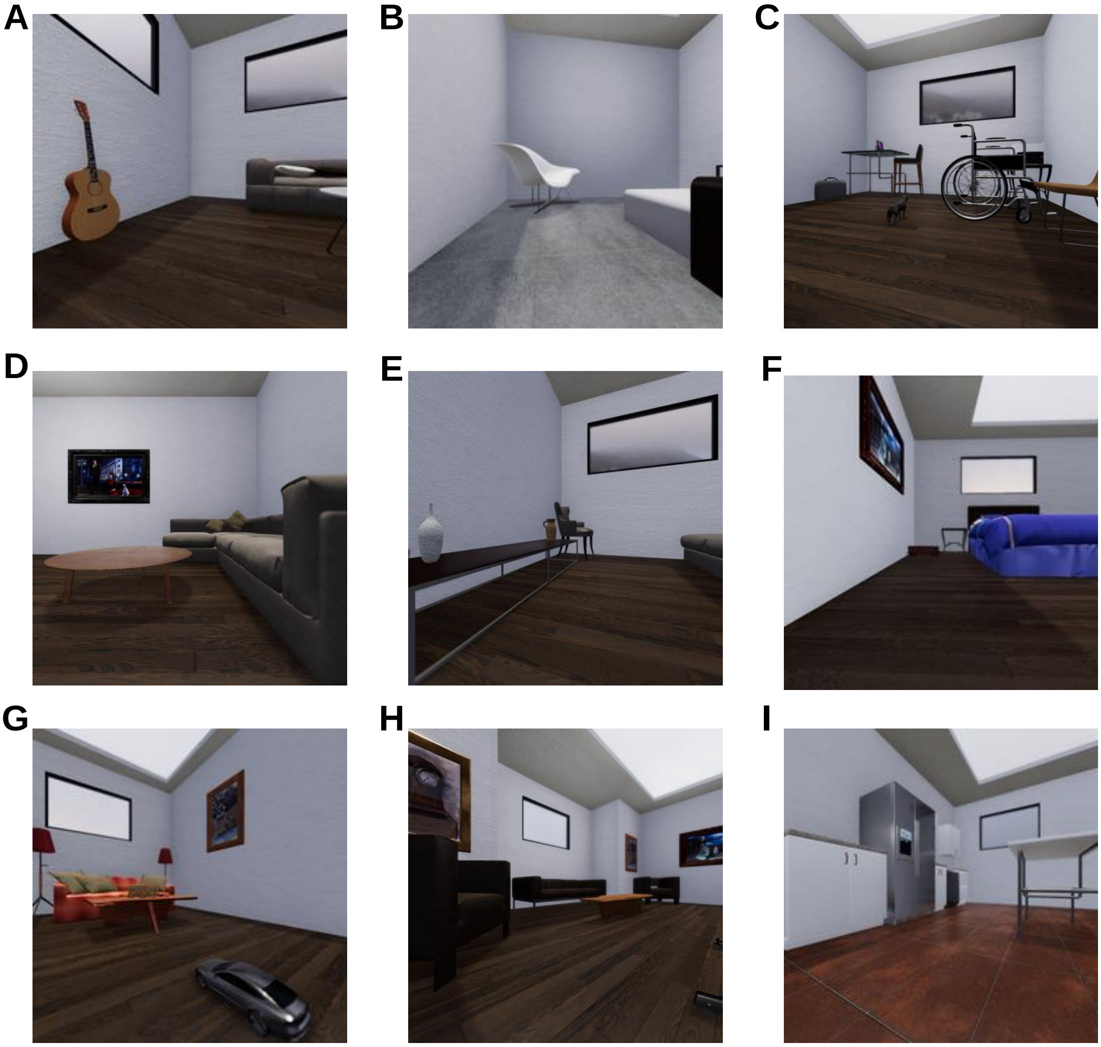

Figure S2: Nine rooms in the Apt environment
Letters A-I represent the rooms 0 through 8 in order.

Room label	x-axis boundary	y-axis boundary	Images
0	(-10.6, -7.2)	(1.45, 4.80)	680
1	(-7.2, -3.1)	(1.45, 4.80)	659
2	(-3.1, 0.8)	(1.45, 4.80)	1312
3	(0.8, 6.8)	(1.45, 4.80)	1295
4	(6.8, 9.8)	(1.45, 4.80)	749
5	(-10.6, -6,2)	(-5.70, -0.18)	1333
6	(-6.2, -3.1)	(-5.70, -0.18)	794
7	(-3.1, 3.3)	(-5.70, -0.18)	1269
8	(3.3, 9.8)	(-5.70, -0.18)	1764

Table S2: **The boundaries of the nine rooms in the Apt environment and the number of samples for each room**
The coordinate ranges are measured in the ThreeDWorld virtual environment.

Learning rate comparison

In our pretext training, we adopted a learning rate of 0.3 instead of the suggested 0.015 from MoCo V2. This adjustment was made based on its improved overall accuracy on our datasets. Table S3 shows the results of MoCo V2 with different learning rates when trained on the House100K dataset.

Learning	Pretext Task		Downstream ImageNet Classification	
rate	training loss	accuracy	Training loss	Test accuracy (%)
0.015	3.73	73.45	209.25	7.61
0.3	4.43	82.11	4.71	17.36

Table S3: **Comparison results of MoCo V2 with two different learning rates trained on the House100K dataset**

Details of the implementation of ESS-MB on other models

SimCLR[S1] is a popular contrastive learning model in which the positive pair of an augmented view is itself. Negative pairs are other augmented samples from the same batch. Our ESS-MB on SimCLR found positive samples from the same batch according to spatial information. All parameters were the same as those in SimCLR. We ran the experiment on a single GPU for 200 epochs as suggested by the code.

DCL[S2] removes the positive pairs' effect on the denominator of InfoNCE loss. We implemented the updated loss function based on our original ESS-MB model for both DCL and ESS-MB with DCL. All the parameters were the same as the ESS-MB on MoCo.

CLSA[S3] categorizes augmentation operations into 'strong' and 'weak' and tries to align the feature distance distribution of views derived from these two augmentation types when finding the positive pairs from the weak augmented samples simultaneously. CLSA inherits the structure of MoCo. Based on the implementation of CLSA, our approach found positive pairs of an augmented view from the dictionary. In our experiment, we kept the hyperparameters of CLSA but modified the dictionary size and learning rate to match our original ESS-MB.

NNCLR[S4] computes similarity according to the proximity within a latent space generated by the encoder to contrastively learn representations from unlabeled images. The Lightly package was used to run NNCLR simulations with the ResNet-18 backbone. To ensure a fair comparison, we switched the backbone of ESS-MB on MoCo to Resnet-18 and trained both models for 200 epochs in the pretraining phase.

MoCo V3[S5] applied the contrastive learning structure to the Vision Transformer[S6] backbone. The dictionary size is set to 4096 to align with the threshold of ESS-MB approach. We run both models for 200 epochs in the pretraining with 256 batch size.

Assessing the clustering of learned features

After training on the datasets, we applied t-SNE[S7] on the features for a subset of images from the corresponding datasets to determine whether the training produced clustering of features from spatially proximal images. We randomly selected approximately 10,000 images that were inside the room boundaries and took the features from the fully trained ResNet model as input to the t-SNE. In the resulting t-SNE space, each image's features were labeled with a number (and a corresponding color), indicating the room of its origin.

The t-SNE visualizations generated for both the baseline and ESS-MB models trained on House and Apt environments are shown in Figures S3 and S4, respectively. Furthermore, we used the Silhouette Coefficient,[S8] Calinski-Harabasz index,[S9] and Davies-Bouldin index[S10] as metrics to assess the clustering quality of the t-SNE outputs. These results are shown in Table S4. Both results show a model trained on the more extensive House100K dataset exhibited a stronger capability in distinguishing features generated from different rooms compared to the one trained on the smaller House14K dataset. For all the models trained on three datasets, ESS-MB exhibited reduced clustering relative to the baseline model. This might be attributed to the ESS-MB training approach, which tends to group features of spatially proximal images together, regardless of room boundaries. In contrast, the baseline MoCo model relies only on instance discrimination. As a result, cluster boundaries for nearby locations in adjacent rooms would not be as distinct with ESS-MB.

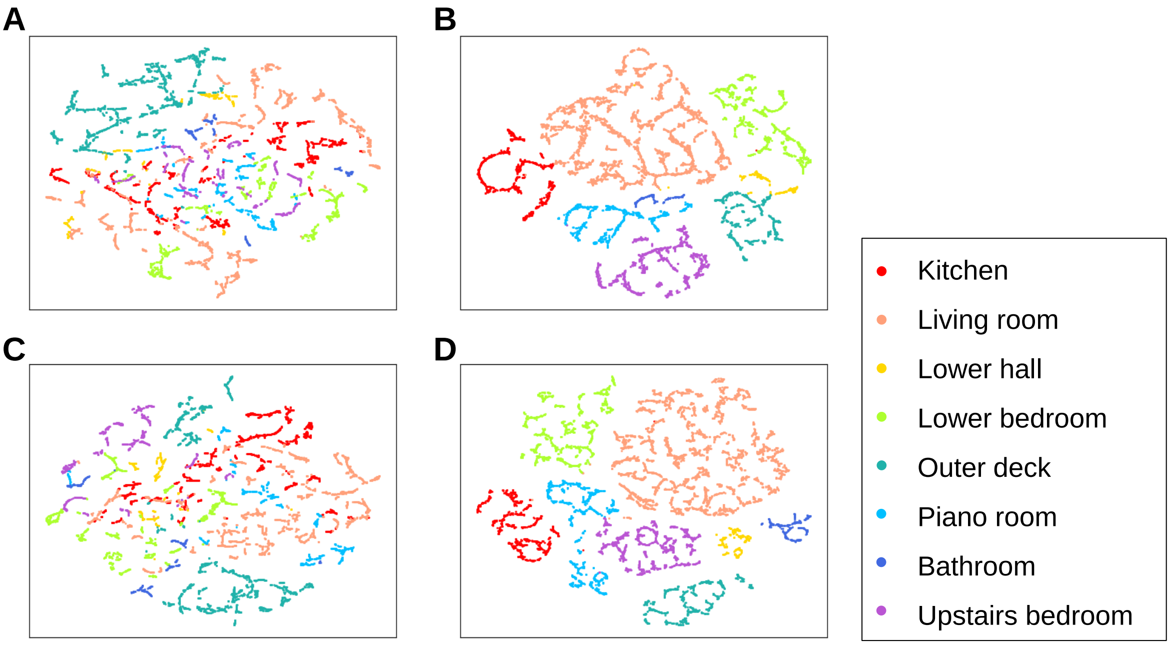

Figure S3: The t-SNE results of the learned features in the House environment
(A) ESS-MB on House14K. (B) ESS-MB on House100K. (C) Baseline on House14K. (D) Baseline on House100K.

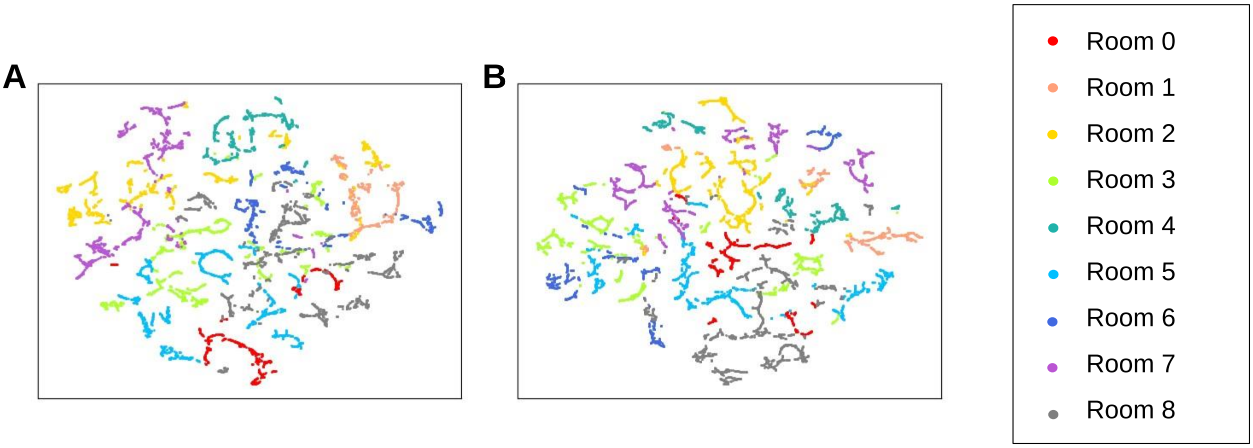

Figure S4: The t-SNE results of the learned features in the Apt environment
(A) ESS-MB on Apt14K. (B) Baseline on Apt14K.

Pretext dataset	Model	Silhouette ↑	CH index ↑	DB index ↓
House100K	Baseline	0.2394	4538.16	0.8552
House100K	ESS-MB	0.1437	2393.92	1.8488
House14K	Baseline	-0.0548	1187.78	5.5367
House14K	ESS-MB	0.0972	1549.44	9.8386

Table S4: **Evaluation of the learned features**
CH and DB stand for Calinski-Harabasz and Davies-Bouldin indices, respectively. An upward arrow indicates that a higher value for the respective index denotes more effective clustering, while a downward arrow implies the reverse.

References

[S1] Chen, T., Kornblith, S., Norouzi, M., and Hinton, G. (2020). A simple framework for contrastive learning of visual representations. In Proc. Int. Conf. Machine Learning, pp. 1597–1607. JMLR.org volume 119. Https://doi.org/10.5555/3524938.3525087.

[S2] Yeh, C.-H., Hong, C.-Y., Hsu, Y.-C., Liu, T.-L., Chen, Y., and LeCun, Y. (2022). Decoupled contrastive learning. In Proc. European Conf. Computer Vision, pp. 668–684. Springer. https://doi.org/10.1007/978-3-031-19809-0_38.

[S3] Wang, X., and Qi, G.-J. (2022). Contrastive learning with stronger augmentations. IEEE Trans. Pattern Anal. Mach. Intell. *45*, 5549–5560. https://doi.org/10.1109/TPAMI.2022.3203630.

[S4] Dwibedi, D., Aytar, Y., Tompson, J., Sermanet, P., and Zisserman, A. (2021). With a little help from my friends: Nearest-neighbor contrastive learning of visual representations. In Proc. IEEE/CVF Int. Conf. on Computer Vision, pp. 9588–9597. IEEE. https://doi.org/10.1109/ICCV48922.2021.00945.

[S5] Chen, X., Xie, S., and He, K. (2021). An empirical study of training self-supervised vision transformers. In Proc. IEEE/CVF Int. Conf. Computer Vision, pp. 9640–9649. https://doi.org/10.1109/ICCV48922.2021.00950.

[S6] Dosovitskiy, A., Beyer, L., Kolesnikov, A., Weissenborn, D., Zhai, X., Unterthiner, T., Dehghani, M., Minderer, M., Heigold, G., Gelly, S. et al. (2021). An image is worth 16x16 words: Transformers for image recognition at scale. arXiv preprint arXiv . https://doi.org/10.48550/arXiv.2104.02057.

[S7] Hinton, G. E., and Roweis, S. (2002). Stochastic neighbor embedding. In Adv. Neural Inf. Process. Syst., p. 857–864. MIT Press volume 15. https://dl.acm.org/doi/abs/10.5555/2968618.2968725.

[S8] Rousseeuw, P. J. (1987). Silhouettes: A graphical aid to the interpretation and validation of cluster analysis. J. Comput. Appl. Math. *20*, 53–65. https://doi.org/10.1016/0377-0427(87)90125-7.

[S9] Caliński, T., and Harabasz, J. (1974). A dendrite method for cluster analysis. Commun. Stat. Theory Methods *3*, 1–27. https://doi.org/10.1080/03610927408827101.

[S10] Davies, D. L., and Bouldin, D. W. (1979). A cluster separation measure. IEEE Trans. Pattern Anal. Mach. Intell. *PAMI-1*, 224–227. https://doi.org/10.1109/TPAMI.1979.4766909.
